# Factors associated with elevated Per- and Polyfluoroalkyl substances serum levels in older adults

**DOI:** 10.1016/j.ahr.2022.100086

**Published:** 2022-06-21

**Authors:** Emmanuel Obeng-Gyasi

**Affiliations:** ^a^Department of Built Environment, North Carolina Agricultural and Technical State University, USA; ^b^Environmental Health and Disease Laboratory, North Carolina Agricultural and Technical State University, USA

**Keywords:** Per- and Polyfluoroalkyl substances, Well water, Education, Income, Ethnicity, Water source

## Abstract

Per- and Polyfluoroalkyl substances (PFAS) are highly persistent synthetic chemicals that have been produced for more than seven decades. This study examined 6,018 eligible older adults (aged≥ 60 years) using the National Health and Nutrition Examination Survey (NHANES), to assess seven PFAS by sociodemographic and water source data to (a) determine factors most linked with elevated PFAS levels and (b) evaluate the differences by race and ethnicity. The results of this study indicated that among older adults, PFAS levels were more elevated in men than women (*p < 0.05)*, non-Hispanic Blacks than other ethnicities *(p < 0.05)*, among those using well water (*p < 0.05)*, and those with lower education (*p < 0.05)*. Income was not a significant factor among this group. These findings offer insight into the factors associated with elevated PFAS levels in older adults. With this knowledge, it is necessary to target education about PFAS among the most vulnerable.

## 1. Introduction

Per- and Polyfluoroalkyl substances (PFAS) are persistent organic pollutants that have been widely used in consumer products for more than seven decades. PFAS are amphiphilic and have strong water and oil repellent properties, they are widely used in many applications such as food packaging, firefighting foams, and some household items [1]. Household exposure can come from dust, drinking water, food, and soil [2]. Due to extensive exposure, measurable PFAS can be found in the blood samples in a significant proportion of the populations worldwide [3]. PFAS can bring forth varying adverse health effects depending on the route of exposure, the magnitude, and the duration [4]. In addition, individual-level factors such as ethnicity, age, sex, genetic predisposition, and current health status potentially influence the adverse health outcomes [5]. Many PFAS are able to cross the placental barrier and enter breast milk [6], with breastfeeding adversely altered by PFAS exposure [7]. Exposure to PFAS particularly harms children with their hand-to-mouth behavior allowing for increased exposure [8]. In adults, PFAS affects several biological systems [9,10]. Owing to PFAS environmental and biological persistence and their effects on human health, it is imperative to understand the factors most associated with elevated levels of PFAS in populations. This is especially the case for older adults who have accumulated PFAS over their life to the potential detriment of their health. For example, the half-life for some PFAS can range from one to five years meaning their effects can cumulatively damage molecular processes in the body [11]. This study explored sociodemographic and water source data of 7 PFAS in older adults to identify factors associated with elevated levels of PFAS in older adults. PFAS exposure levels were assessed using PFAS serum levels as this can serve as a proxy for actual exposure.

## 2. Methods

### 2.1. Study population

PFAS were explored in 6,018 US older adults using National Health and Nutrition Examination Survey (NHANES) 2007–14 data. The inclusion criteria were adults aged ≥ 60 years, having biomarkers of interest present, and having sociodemographic and water source data present. The sociodemographic factors were ethnicity, gender, education, and income. The biomarkers of interest were the PFAS. Specifically, seven PFAS including PPerfluorononanoic acid (PFNA), perfluorooctanoic acid (PFOA), perfluorooctane sulfonic acid (PFOS), and perfluorodecanoic acid (PFDE), perfluorohexane sulfonate (PFHxS), perfluorooctane sulfonamide (PFOSA), and perfluoroundecanoate (PFUA) were explored. The water source data refer that participants got their water from a public/private well or from a public/private water company.

NHANES is a multistage, stratified, complex survey, designed to examine the health and nutritional status of a nationally representative sample of non-institutionalized individuals. NHANES uses a complex survey design with unequal probabilities for selection, complex estimation methods are required to generate population estimates. In the final dataset, weights account for the unequal probability of selection and nonresponse and include poststratification to US Census estimates of the general population. The NHANES public-use dataset comprises individual sample weights for the interview and mobile examination center (MEC) examination.

Detailed descriptions of PFAS laboratory analytic methods used have been published elsewhere [12].

### 2.2. Statistical analysis

Geometric mean serum levels were calculated for each of the seven compounds. Geometric means and p values were calculated using the stratum, cluster, and subsample weights provided by NHANES. Mean differences between subgroups were assessed. STATA’s post-estimation command “lincom” was used to make the aforementioned comparisons between subgroups. Analysis only proceeded on available data. Multiple linear regression analysis, which included water source, gender, ethnicity, education, and income as predictor variables of interest for PFAS was performed to determine which factors were significantly associated with various PFAS in older adults. Stata SE/15.0 (StataCorp, College Station, TX, USA) was used for the analysis of the data, as the software allowed for the analysis of the complex study design and the weights designated by NHANES. The *Stata* lincom command allowed for the computation of a two tailed p-value with a *p <* 0.05 considered statistically significant.

## 3. Results

A summary of demographic factors of the studied population can be found in Table 1. The mean age was 70 years old with 51%being females. The population was predominantly Non-Hispanic White (79%), with most incomes (57.7%) falling in the 20,000–99,000-dollar range, and a significant number having a high school diploma or less and no college (48%).

The results of PFAS by ethnicity, gender, water source, education and income were explored in Tables 2–6. Table 2 first explores mean differences of the seven PFAS of interest by gender and ethnicity (N=1726), then Table 3 by water source and ethnicity (N=1239), then Table 4 by education (N=1726), then Table 5 by income (N=1720), finally in Table 6 a multiple linear regression is performed with all of these variables of interest. The ethnicities examined were Mexican-American, Other Hispanic, Non-Hispanic White, and Non-Hispanic Black. The trend indicated that the non-Hispanic Black men tended to have significantly (*p <* 0.05) higher levels of the PFAS of interest compared to other ethnicities and females. PFAS serum levels were evaluated via education and income levels. The results generally demonstrated that older adults with higher education levels have lower PFAS serum levels (*p <* 0.05). No statistically significant differences across the various income groups were observed (*p >* 0.05). The source of tap water in the house was explored by ethnicity to see whether using water from a private or public water company (WC), or private or public well (WW) impacted PFAS serum levels. The results indicated that non-Hispanic Black older adults using WW tended to have higher levels of PFAS. Multiple linear regression analysis with water source, gender, ethnicity, education and income as predictor variables of interest for PFAS indicated that gender and ethnicity were statistically significant factors in exposure to various PFAS, as shown in Table 6.

## 4. Discussion

Many PFAS bioaccumulate in tissues and have elimination half-lives of several years in humans. Indeed, the carbon-fluorine bonds responsible for repellent effects are highly chemically and thermally stable, making PFAS remarkably resilient and consequently difficult to degrade and/or destroy. It was thus critical to investigate which demographic and water source data were most significantly associated with elevated PFAS levels in older adults to understand who is being exposed to these toxic chemicals.

This study found that PFAS serum levels among older adults varied by ethnicity, with non-Hispanic Blacks having the highest levels. This may be explained by several factors such as consuming prepared food in coated cardboard containers, having stain-resistant carpet or furniture, and living in a city served by a PFAS-contaminated water supply [12]. More studies are needed on the food consumption behaviors, housing characteristics, and water source data of populations at risk for elevated PFAS serum levels to pinpoint the factors associated with differential exposure to various PFAS. In addition, further exploration of these demographics in different geographical locations may offer more insight into PFAS serum levels by race. Finally, examining factors such as excretion rate, and other toxicokinetic considerations by demographic characteristics may help close the gap in knowledge on these findings.

This study found that gender was a significant factor in PFAS serum levels, with men having higher levels than women. Other studies have found gender to be important for some exposure-health associations [13] with factors such as obesity potentially a significant factor in differential of PFAS exposure. These differences may be due to occupation [14], with occupations such as construction laborers, firefighting, automotive technicians, and mechanics dominated by men and more likely to expose workers to PFAS.

Factors such as breastfeeding and menstruation may also explain differences in body burden, with breastfeeding and menstruation serving as a critical elimination route for some PFAS [15], though this may not apply to the age group in this study [16]. However, these factors over the lifespan would still have an impact on this group, this will become clearer with further studies on the toxicokinetic and toxicodynamic properties of various PFAS [17].

This study found that education was a factor in PFAS serum levels. Education has been noted as a critical factor in exposure in other studies [18]. The differences in PFAS serum levels by education may be related to factors such as occupation which is often tied to education. It may also be related to PFAS effects on neurocognition as PFAS has been shown to have neurotoxic effects in some studies [19].

No significant relationship between income and PFAS serum levels was observed. Due to potential retirement among the age group in this study, income is not a perfect indicator. To further explore this, more data would be needed on occupation prior to retirement and geographical location. Other studies with adults younger than those in this study, found higher income is associated with higher internal exposure to some PFAS, with a doubling of the income increasing internal exposure 10–14% [20].

This study found that water source and ethnicity together were significant factors in PFAS serum levels. The higher levels of PFAS among non-Hispanic Blacks who got their water from wells potentially speaks to race being a strong determinant for the access to safe water.

Limitations of this study included the cross-sectional design, which could limit causal inference since it is difficult to ascertain when exposure and outcome occurred in the time continuum.

## 5. Conclusions

PFAS serum levels in older US adults are different across gender, education, source of water, and ethnicity. Future works could explore these areas in detail like why older non-Hispanic Blacks have higher serum PFAS levels.

## Figures and Tables

**Table 1:** Demographic characteristics of the study participants.

Characteristics	
N	6,018
Female, n (%):	3069 (51.0)
Age, mean (SE)	70.0 (0.15)
Ethnicity, n (%)	
Mexican American	233 (3.9)
Other Hispanic	211 (3.5)
Non-Hispanic White	4,728 (79.0)
Non-Hispanic Black	533 (8.9)
Other Race	313 (5.2)
Income range, n (%)	1054 (17.6)
$ 0 to $19,999	3453 (57.7)
$ 20,000 to $ 99,999	939 (16.0)
>$100,000	532 (8.90)
Other	
Education,n (%)	619 (10.0)
Less Than 9th Grade	752 (13.0)
9–11th Grade (Includes 12th grade with no diploma)	1480 (25.0)
High School Grad/GED or Equivalent	1620 (27.0)
Some College or AA degree	1537 (26.0)
College Graduate or above	0 (0)
Other	

**Table 2:** PFAS serum levels stratified by ethnicity and sex.

Ethnicities	Gender	PFNAng/mL		PFOAng/mL		PFOSng/mL		PFDEng/mL		PFHxSng/mL		PFOSAng/mL		PFUAng/mL	
		Mean	SE	Mean	SE	Mean	SE	Mean	SE	Mean	SE	Mean	SE	Mean	SE
Total Population	Male	1.7	0.12	4.3	0.16	21.0	1.2	0.41	0.03	3.0	0.22	0.46	0.03	0.31	0.02
	Female	1.6	0.11	4.1	0.14	15.0	0.64	0.37	0.02	2.7	0.11	0.44	0.02	0.26	0.02
Mexican American	Male	1.2	0.10	3.2	0.20	15.0	1.7	0.35	0.05	2.4	0.32	0.33	0.04	0.17	0.02
	Female	1.3	0.08	3.6	0.20	12.0	0.91	0.35	0.04	2.3	0.28	0.27	0.03	0.17	0.03
Other Hispanic	Male	1.3	0.11	3.6	0.34	13.0	1.3	0.31	0.03	2.5	0.27	0.27	0.04	0.23	0.02
	Female	1.3	0.70	3.6	0.37	11.0	1.2	0.25	0.02	2.3	0.43	0.24	0.04	0.20	0.02
Non-Hispanic White	Male	1.6	0.11	4.4	0.19	20.0	1.0	0.35	0.04	3.0	0.27	0.48	0.03	0.27	0.02
	Female	1.5	0.12	4.1	0.17	15.0	0.77	0.33	0.02	2.7	0.13	0.46	0.03	0.23	0.20
Non-Hispanic Black	Male	2.1	0.20	4.5	0.26	29.0	2.6	0.62	0.06	3.6	0.34	0.58	0.06	0.49	0.06
	Female	2.5	0.46	4.0	0.34	21.0	2.0	0.58	0.06	2.6	0.18	0.53	0.09	0.47	0.05

**Table 3:** PFAS serum levels stratified by ethnicity and water source.

Ethnicities	Water Source	PFNAng/mL		PFOAng/mL		PFOSng/mL		PFDEng/mL		PFHxSng/mL		PFOSAng/mL		PFUAng/mL	
		Mean	SE	Mean	SE	Mean	SE	Mean	SE	Mean	SE	Mean	SE	Mean	SE
Mexican American	WC	1.3	0.08	3.8	0.17	15.0	1.1	0.39	0.03	2.7	0.34	0.32	0.04	0.19	0.02
	WW	1.4	0.48	4.2	1.2	13.0	2.0	0.35	0.08	2.6	0.62	0.45	0.02	0.16	0.03
Other Hispanic	WC	1.4	0.12	4.2	0.42	15.0	1.6	0.31	0.03	2.7	0.34	0.38	0.04	0.23	0.02
	WW	0.98	0.27	3.3	0.35	11.0	1.5	0.27	0.09	2.8	0.64	0.30	0.03	0.18	0.06
Non-Hispanic White	WC	1.6	0.18	4.8	0.20	19.0	1.3	0.35	0.02	3.0	0.17	0.49	0.03	0.25	0.03
	WW	1.8	0.25	4.7	0.23	20.0	0.83	0.41	0.06	2.9	0.18	0.63	0.08	0.31	0.05
Non-Hispanic Black	WC	2.3	0.20	4.8	0.22	28.0	2.3	0.69	0.05	3.3	0.24	0.61	0.09	0.53	0.04
	WW	3.9	1.4	5.0	1.2	33.0	7.9	0.99	0.34	2.9	0.33	0.40	0.08	0.85	0.31

**Table 4:** PFAS serum levels stratified by education.

Education	PFNAng/mL		PFOAng/mL		PFOSng/mL		PFDEng/mL		PFHxSng/mL		PFOSAng/mL		PFUAng/mL	
	Mean	SE	Mean	SE	Mean	SE	Mean	SE	Mean	SE	Mean	SE	Mean	SE
less than a 9th grade education	1.6	0.20	3.7	0.21	18.0	1.7	0.44	0.04	2.5	0.20	0.42	0.04	0.33	0.03
High school grad or GED equivalent	1.8	0.20	4.6	0.24	19.0	1.3	0.38	0.33	3.1	0.38	0.46	0.04	0.27	0.03
College graduate or above	1.6	0.11	4.1	0.19	15.0	0.90	0.35	0.02	2.7	0.19	0.38	0.03	0.28	0.02

**Table 5:** PFAS serum levels stratified by income.

Income	PFNAng/mL		PFOAng/mL		PFOSng/mL		PFDEng/mL		PFHxSng/mL		PFOSAng/mL		PFUAng/mL	
	Mean	SE	Mean	SE	Mean	SE	Mean	SE	Mean	SE	Mean	SE	Mean	SE
< $20,000	1.3	0.27	3.4	0.59	14.0	2.6	0.35	0.04	3.7	1.4	0.77	0.23	0.25	0.10
$20,000-$99,999	1.5	0.12	4.1	0.44	17.0	1.6	0.41	0.04	2.6	0.38	0.34	0.04	0.32	0.04
>$100,000	1.6	0.13	3.8	0.19	16.0	1.3	0.39	0.04	2.7	0.20	0.38	0.04	0.31	0.03

**Table 6:** Multiple Linear Regression analysis of PFAS serum levels and water source, sex, ethnicity, education, and income.

Regression Analysis	PFNAng/mL	PFOAng/mL	PFOSng/mL	PFDEng/mL	PFHxSng/mL	PFOSAng/mL	PFUAng/mL
Water Source	-	-	-	-	-	-	-
Gender	B = −0.091*P* = 0.027	-	B = −0.295, *p <* 0.001	-	-	-	B = −0.121 *P* = 0.011
Ethnicity	B = 0.126*P* = 0.003	-	B = 0.141*P* = 0.001	B = 0.196*P* = 0.001	-	-	B = 0.276*p* < *0.001*
Education	-	-	-	-	-	-	-
Income	-	-	-	-	-	-	-
